# Supplementary material for: Induced Periosteum-Mimicking Membrane with Cell Barrier and Multipotential Stromal Cell (MSC) Homing Functionalities
Source: Int J Mol Sci. 2020 Jul 23;21(15):5233. doi: 10.3390/ijms21155233 (PMC7432450; doi:10.3390/ijms21155233)
Supplement: Supplementary file 1 [file ijms-21-05233-s001.pdf]

Supplementary Material

Supplementary Table 1: Details of human samples of periosteum and induced membrane. * Donors shown in Figure 1

	Donor Details (gender, age – years)	Harvested From	Time Since Injury (weeks)	PMMA Cement Spacer *in situ* (weeks)
**Periosteum**	Male, 23	Femur	57	-
	Male, 35	Humerus	108	-
	Male, 47	Femur	19	-
	Male, 61	Iliac Crest	0.5	-
	Female, 74	Femur	49	-
	Female, 80*	Femur	17	-
**Induced Membrane**	Male, 32	Femur	-	14
	Female, 34	Tibia	-	8
	Female, 40	Ulna	-	7
	Male, 44	Femur	-	3
	Female, 47	Tibia	-	7
	Male, 58*	Femur	-	17

Histological Staining

Haematoxylin and Eosin

H&E dyes nuclei (haematoxylin) and cytoplasm or ECM structures (eosin). Sections were stained for 2 mins in haematoxylin, rinsed in Scott's Tap Water to remove excess dye and then placed in eosin for 2 mins.

Picro Sirius Red

Picro Sirius Red stains for collagen and nuclei (Wiegert's haematoxylin). Slices were stained in haematoxylin for 8 mins followed by a 10 min wash in tap water. Sections were then placed in PSR for 1 h before being washed in acidified water (0.5% glacial acetic acid, in dH_2O).

Confocal Microscopy Staining Protocol

Cell Attachment Assay

Samples were stained with DAPI and Phalloidin-FITC, attached cells were permeabilised using 0.1% Tween for 10 min, followed by staining with 0.1% Phalloidin-FITC (15 mins, dark) and DAPI (0.1 %, 1 h, dark), with PBS washes between staining. Stained samples were mounted onto slides using VECTASHIELD® Vibrance™ Antifade Mounting Medium (Vector Labs) and imaged. To assess cellular alignment five images per experimental time condition were taken. DAPI images were made binary in ImageJ, individual objects (nucleus) were counted and measured for alignment.

Modified Transwell Barrier Assay

A drop of Prolong Gold Antifade DAPI (ThermoFisher) was added on top of the sample and then covered with a coverslip and left to cure overnight, followed by sealing with clear nail varnish. The top and bottom of the membranes were imaged (n=3) using confocal microscopy.
